# Conscious and unconscious processes in cognitive control: a theoretical perspective and a novel empirical approach

**DOI:** 10.3389/fnhum.2012.00199

**Published:** 2012-07-04

**Authors:** Guillermo Horga, Tiago V. Maia

**Affiliations:** ^1^Department of Psychiatry, Columbia University, New YorkNY, USA; ^2^New York State Psychiatric Institute, New YorkNY, USA; ^3^Instituto de Medicina Molecular and Instituto de Fisiologia, Faculdade de Medicina da Universidade de LisboaLisboa, Portugal

**Keywords:** cognitive control, conflict monitoring, conscious, medial prefrontal cortex, prefrontal cortex, subliminal priming, unconscious

## Abstract

Controlled processing is often referred to as “voluntary” or “willful” and therefore assumed to depend entirely on conscious processes. Recent studies using subliminal-priming paradigms, however, have started to question this assumption. Specifically, these studies have shown that subliminally presented stimuli can induce adjustments in control. Such findings are not immediately reconcilable with the view that conscious and unconscious processes are separate, with each having its own neural substrates and modus operandi. We propose a different theoretical perspective that suggests that conscious and unconscious processes might be implemented by the same neural substrates and largely perform the same neural computations, with the distinction between the two arising mostly from the quality of representations (although not all brain regions may be capable of supporting conscious representations). Thus, stronger and more durable neuronal firing would give rise to conscious processes; weaker or less durable neuronal firing would remain below the threshold of consciousness but still be causally efficacious in affecting behavior. We show that this perspective naturally explains the findings that subliminally presented primes induce adjustments in cognitive control. We also highlight an important gap in this literature: whereas subliminal-priming paradigms demonstrate that an unconsciously presented prime is sufficient to induce adjustments in cognitive control, they are uninformative about what occurs under standard task conditions. In standard tasks, the stimuli themselves are consciously perceived; however, the extent to which the processes that lead to adjustments in control are conscious or unconscious remains unexplored. We propose a new paradigm suitable to investigate these issues and to test important predictions of our hypothesis that conscious and unconscious processes both engage the same control machinery, differing mostly in the quality of the representations.

Humans and other animals adjust their behavior flexibly in the pursuit of goals. Cognitive control mechanisms are the set of processes that allow for such flexible adjustments. For instance, cognitive control is necessary to override automatic or habitual responses when they conflict with current goals—a process that has been long studied through tasks such as the Stroop or Simon Spatial Incompatibility tasks. The common ground of these tasks is the experimental induction of *conflict* between a prepotent response and a weaker response that is correct according to the task goal. Often this conflict is induced by different features of the same stimulus (e.g., the location of an arrow relative to the midline and the direction in which the arrow is pointing), with one of the features stimulating a prepotent response tendency (e.g., a left response to an arrow presented to the left of the midline) and the other feature indicating the response that is correct according to the task goal (e.g., a right response to a right-pointing arrow, even if it is presented to the left of the midline). To resolve the conflict induced by these *incongruent* stimuli, cognitive control mechanisms provide top-down biases that facilitate the goal-directed response over the more automatic one (Miller and Cohen, 2001; Maia and Cleeremans, 2005). Conscious will has been classically assumed to govern this type of controlled processing: i.e., voluntary, conscious processes would be required to select the goal-directed response.

The detection and resolution of conflict, importantly, are nonstatic processes that depend heavily on the task context. An effect common to all conflict tasks, the *conflict-adaptation* effect, illustrates this contextual dependency. Conflict adaptation is the improvement in the resolution of conflict following the experience of conflict. Such adaptation typically occurs on a trial-to-trial basis (Gratton et al., 1992; Egner, 2007) but it also arises on a blockwise basis (Tzelgov et al., 1992; Carter et al., 2000). The description of the conflict-adaptation effect prompted the development of influential models of cognitive control that accounted for both conflict resolution and its contextual adaptability. The influential conflict-monitoring model (Botvinick et al., 2001), for example, proposed that a monitoring system, putatively located in the anterior cingulate cortex (ACC) and activated by conflict, signaled to the prefrontal cortex (PFC) the need to further boost top-down biases that enhanced task-relevant information-processing pathways. As a result, task-relevant responses would be facilitated following conflict, and conflict resolution would, therefore, be more efficient, thereby explaining the conflict-adaptation effect. Despite some findings that are at odds with this model and the existence of several competing theories (Holroyd and Coles, 2002; Brown and Braver, 2005; Critchley, 2005; Carter and van Veen, 2007), substantial evidence supports several aspects of this model (MacDonald et al., 2000; Botvinick et al., 2001; Kerns et al., 2004; Kerns, 2006; Carter and van Veen, 2007).

The conflict-monitoring model does not itself address the potential role of consciousness in controlled processing [despite the close relation between similar cognitive-control models and models of consciousness (Maia and Cleeremans, 2005)]. We suggest, however, that one can take advantage of the model's clearly delineated mechanisms to consider which cognitive control mechanisms might be dependent on conscious processing and which might potentially operate unconsciously. Such an approach allows us to move from simple descriptive questions about the conscious or unconscious correlates of behavior to more detailed questions about the potential implication and roles of conscious and unconscious processing in the mechanisms of cognitive control. For example, we can reformulate the question of whether conflict adaptation requires conscious knowledge to the more mechanistic question of whether the detection of conflict by the ACC and the subsequent strengthening of control by the PFC require conscious knowledge. Importantly, such a reformulation is not merely “cosmetic,” as it raises multiple empirically testable questions about the dependence of the different components of the model on conscious versus unconscious processes. For example, does the detection of conflict by the ACC need to become conscious for conflict adaptation to occur? If so, what level of conscious knowledge is required: explicit knowledge about the preceding conflict or just a vague feeling that performance is not going well? And what is the relationship between ACC activation with conflict and explicit knowledge of conflict? Does the ACC form the core of such knowledge, does it instead receive information about that knowledge from other brain region(s) that modulate its activation, or are the two completely independent? Moving from the detection of conflict by the ACC to the strengthening of control by the PFC, does such strengthening reflect a willful, conscious cognitive act, or is perhaps the order of causality the opposite, with the engagement of PFC giving rise to the “illusion” (Wegner, 2002) of, say, deciding to pay more attention to a given stimulus feature?

Here we will use the term *consciousness* to refer exclusively to the content of conscious representations. As articulated in more detail elsewhere, we take consciousness to be the result of a global constraint satisfaction process in which the winning neuronal coalition determines both accessibility and phenomenal experience (Maia and Cleeremans, 2005). We will center our discussion on whether conscious representations of current events, goals, and contexts are needed for conflict resolution and its contextual adaptation. We will further assume that these conscious representations, in contrast with long-term knowledge that is embedded in synaptic weights, rely on more transient, active representations encoded in the firing patterns of neurons. These active representations, unlike weight-based knowledge, can be accessible to other systems and are thought to be necessary, though not sufficient, for conscious awareness (Maia and Cleeremans, 2005). Even when different active representations originate in the same neuronal ensemble, the *quality* of the representations—i.e., their strength, duration, stability, distinctiveness, etc.,—might render only some of these representations accessible to consciousness. Some brain regions may potentially contribute less or not at all to conscious experience (Godwin et al., in press), so in those regions, even high-quality representations might not lead to conscious awareness. For instance, converging evidence suggests that while perceptual information in the ventral visual stream can become conscious (Doesburg et al., 2009), perceptual representations in the dorsal stream for visuomotor action may not be accessible to consciousness (Goodale and Milner, 1992, 2005). The regions involved in conflict monitoring and cognitive control, however, seem particularly likely to be implicated in conscious awareness (Morsella, 2005).

The core of our hypothesis is that the same types of representations in the same brain regions may give rise to either conscious or unconscious knowledge, depending on the quality of the representation—an idea that is consistent with a variety of lines of evidence, old (Kinsbourne, 1988) and new (Maia and Cleeremans, 2005). Such an effect may be direct, with high-quality representations becoming conscious *per se*, or it may be due to the fact that high-quality representations will have a higher probability of entering the “global workspace” (Baars, 1988; Dehaene et al., 2003) or winning the global constraint-satisfaction competition (Maia and Cleeremans, 2005). Even weak (and therefore unconscious) representations, however, can be causally efficacious in changing neuronal processing downstream (Cleeremans, 2004). Thus, we should not be surprised if unconscious processing—elicited, for example, by the subliminal presentation of stimuli (which simply elicits weaker representations)—produces effects similar to, but weaker than, supraliminal presentation of the same stimuli. This overarching theoretical perspective about the nature of conscious versus unconscious processing also allows us to cast our original questions in even more mechanistic terms, by asking whether active representations of a special quality are required for controlled processes and, if so, which specific control mechanisms require these special representations.

## Subliminal-priming studies of the capability of unconscious processes to influence cognitive control

Subliminal-priming studies (also known as masked-priming studies) have been used to assess whether unconscious processes affect a variety of cognitive, affective, and behavioral processes (Eimer and Schlaghecken, 2003; van den Bussche et al., 2009). In these studies, stimuli are presented very briefly before being masked by another stimulus, so that the initial stimulus remains outside of awareness. Whether this manipulation does indeed render perception of the initial stimulus fully unconscious is not always uncontroversial—for example, at least some of these studies might be underpowered to detect above-chance discrimination of masked primes and thus wrongly assume unconscious perception of those primes (Szczepanowski and Pessoa, 2007). Nonetheless, the subliminal-priming approach is often assumed to indeed make perception of the initial stimulus (the prime) unconscious. For this reason, this approach has been used in conflict tasks to assess whether unconscious processes can affect cognitive control. Using masked and unmasked primes, an early study found that only consciously perceived conflict triggered conflict adaptation (Kunde, 2003). This result was interpreted as proof that only conscious information is used to adjust control. Later reports, however, seem directly at odds with this interpretation. Recent work has shown that both “unconscious errors”—defined as Go trials that followed a masked No-Go cue, but in which participants executed a response—and unconsciously primed conflict induce subsequent adjustments in behavior (Cohen et al., 2009; van Gaal et al., 2010). In particular, even conflict stimuli that are presented subliminally can induce conflict adaptation. These and other findings suggest instead that unconscious processing of information has many complex features that were once thought to be unique to its conscious counterpart (Wokke et al., 2011). Thus, unconscious processing of information seemingly can lead to adjustments in cognitive control. These findings, along with others similarly demonstrating that unconscious processes have many of the characteristics traditionally associated with conscious processes, are fully consistent with our view that the same brain regions can perform the same set of processes when stimulated subliminally and when stimulated supraliminally, with the main difference being the quality and strength of the resulting representations and processing. Further support for our view comes from the finding that the magnitude of the conflict-adaptation effect varies with the masking strength of the conflict-inducing prime: conflict adaptation following conscious primes is considerably greater than conflict adaptation following unconscious primes (van Gaal et al., 2010). In our view, this occurs simply because the subliminal presentation of stimuli does not have sufficient duration to elicit strong and durable neuronal firing, whereas the supraliminal presentation does.

Other studies have exploited both positive and negative effects in subliminal priming. While masked primes initially activate responses associated with the prime, thereby facilitating responses to targets that are compatible with it, at longer delays between prime and target this response facilitation turns into an inhibition (Eimer and Schlaghecken, 1998). A recent study used subliminal presentation of arrow primes (corresponding or non-corresponding with the target arrow) and measured the effect of long and short prime-target intervals on the response to a target arrow flanked by other arrows (congruent or incongruent flankers; Boy et al., 2010). The study showed that prime-induced inhibition at long intervals differentially affected responses to the current target depending on whether the target's flankers were congruent or incongruent with it. When the prime differed from the target, there was almost no additional cost for responses to incongruent as compared with congruent trials. Because subliminal priming interacted with current-trial congruence but not with conflict adaptation (i.e., the effect of unconscious inhibition was the same on incongruent trials preceded by an incongruent trial and on incongruent trials preceded by a congruent trial), the authors argued that unconscious inhibition might separate two types of control processes: a responsive (post-stimulus) control, related to conflict resolution, which might share motor mechanisms with unconscious processes, and a preparatory (pre-stimulus) control linked to conflict adaptation and which is impervious to unconscious inhibition. Although this distinction is appealing, an alternative account of these results is that unconscious inhibition does not affect either pre- or post-stimulus control. The putative effect on post-stimulus control—the abovementioned finding that unconscious inhibition nearly abolished the extra cost for incongruent as compared to congruent trials—can in fact be given a simple explanation: at long intervals, primes that differ from the target inhibit the response tendency to the non-target direction, and therefore flankers that signal that direction have a weaker effect. Thus, there is reduced conflict when an incongruent trial is presented after such primes, and the response to the target becomes easier. Conversely, at short intervals, primes aligned with the target may facilitate reaction times in a nonspecific manner without weakening the effect of flankers, and thus, without reducing conflict. Regardless of the interpretation, since primes in this study were always presented unconsciously and flankers were presented consciously, these results provide additional support for our view that both conscious and unconscious stimulation of response tendencies engage overlapping brain regions and therefore interact with each other.

In summary, putting aside potential sensitivity issues in establishing the chance-level discrimination of masked primes necessary to assume unconscious processing (Szczepanowski and Pessoa, 2007), the subliminal-priming studies reviewed here provide strongly suggestive evidence that information that is unconsciously processed can induce certain events (e.g., conflict or error) that in turn engage control mechanisms.

## Alternative approaches to study unconscious influences on cognitive control

Under our suggestion that conscious and unconscious processes might share common mechanisms and differ mostly in terms of representation quality, unconscious processes would indeed be expected to influence control mechanisms, like their conscious counterparts do (Suhler and Churchland, 2009). Future studies should seek to elucidate whether the quality of representations and the conscious experiences associated with them have an influence on control, and if so, on which components of control. Our prediction is that their influence on control will not be qualitatively different but will be quantitatively stronger than that of unconscious processes, simply because stronger representations—potentially further amplified when they enter consciousness's “global workspace” (Baars, 1988; Dehaene et al., 2003) or become part of the attractor state that solves the global constraint-satisfaction problem (Maia and Cleeremans, 2005)—have greater causal efficacy. We suggest that a sensitive assessment of the conscious knowledge that participants are able to report during a standard conflict task, in parallel with behavioral and imaging measures, would help tackle these issues. Here, we delineate this multimodal approach.

### Consciousness and the subcomponents of cognitive control

As mentioned earlier, influential models of cognitive control have successfully accounted for behavioral effects in conflict tasks by incorporating several interacting neural components. In particular, the conflict-monitoring model accounts for conflict adaptation via a projection from a conflict-monitoring unit to a control unit, thereby allowing the occurrence of conflict on incongruent trials to trigger adjustments in control that improve performance on subsequent trials (Botvinick et al., 2001). The conflict-monitoring unit and the control unit are hypothesized to map onto the dorsal anterior cingulate cortex (dACC) and the dorsolateral prefrontal cortex (DLPFC), respectively. Thus, the model predicts that dACC conflict-related activity on the current trial predicts both greater DLPFC activity and greater adjustments in behavior on the subsequent trial—a prediction that has been confirmed empirically (Kerns et al., 2004). Research in nonhuman primates has added to this picture of how subcomponents of cognitive control interact. In particular, neuronal recordings in behaving monkeys have demonstrated that activity during inter-stimulus intervals in a population of neurons in the principal sulcus represents the previous trial's conflict (Mansouri et al., 2007). Furthermore, lesions to this region impair behavioral adjustments following conflict. These findings led to an extension of the conflict-monitoring model that posits that a mnemonic system encoding a representation of previous conflict (before the presentation of the following stimulus) is responsible for adjustments in behavior in the subsequent trial (Mansouri et al., 2009).

Using functional magnetic resonance imaging (fMRI), we recently identified a neural system, comprising the rostral dorsomedial prefrontal cortex (DMPFC) and portions of the DLPFC, that encodes the history of previously experienced conflict during inter-stimulus intervals in humans (Horga et al., 2011). We also demonstrated that this system reflects not only conflict in the immediately preceding trial but also the longer history of conflict in several preceding trials. This system interacted with a second system that was engaged by conflict in the current trial, an interaction that predicted trial-to-trial behavioral adjustments prompted by conflict (i.e., adjustments in the response to the current conflict trial relative to the response to the preceding conflict trial). In our study, inter-stimulus activation in the DMPFC-DLPFC had control-like features: it tracked conflict history and subsequently modulated other brain regions in a top-down manner. Unfortunately, this study was not designed to evaluate the degree to which conscious knowledge of previous conflict history was related to the activation of this control system or to the behavioral adjustments that ensued.

We interpreted the information encoded in the inter-stimulus DMPFC-DLPFC activation as either a memory trace of past conflict or a strategic expectancy. This distinction between a *reactive* memory process that is passively activated by conflict and a *proactive* process that anticipates the occurrence of a certain stimulus type and prepares an optimal action strategy accordingly, respectively, could potentially be important to understand the mechanisms of cognitive control. One way to parse out a purely mnemonic versus an expectancy account is to evaluate whether inter-stimulus activation in the DMPFC-DLPFC system can predict subsequent strategies, specifically certain oculomotor strategies that would only be beneficial if a stimulus of the expected type (e.g., incongruent) appears. Thus, monitoring a strategy such as the focusing of spatial attention—relevant to the resolution of conflict in spatial conflict tasks (Botvinick et al., 2001)—could be a viable way to determine if conflict is expected (whether such expectation is conscious or unconscious). That is, if inter-stimulus activation in the DMPFC-DLPFC system—measured with hemodynamic or electrophysiological imaging—predicted the spatial focusing of attention on the following trial, then this activation would be consistent with an expectancy account. This finding would be particularly compelling if such activation influenced oculomotor strategy, as measured with eye tracking, before an individual has enough time to process the stimulus (and potentially re-adjust the strategy after stimulus presentation), and most importantly, if the oculomotor strategy were specifically beneficial for responding to conflict trials but impaired performance on non-conflict trials.

### Conscious knowledge and conflict adaptation

**Figure 1:**
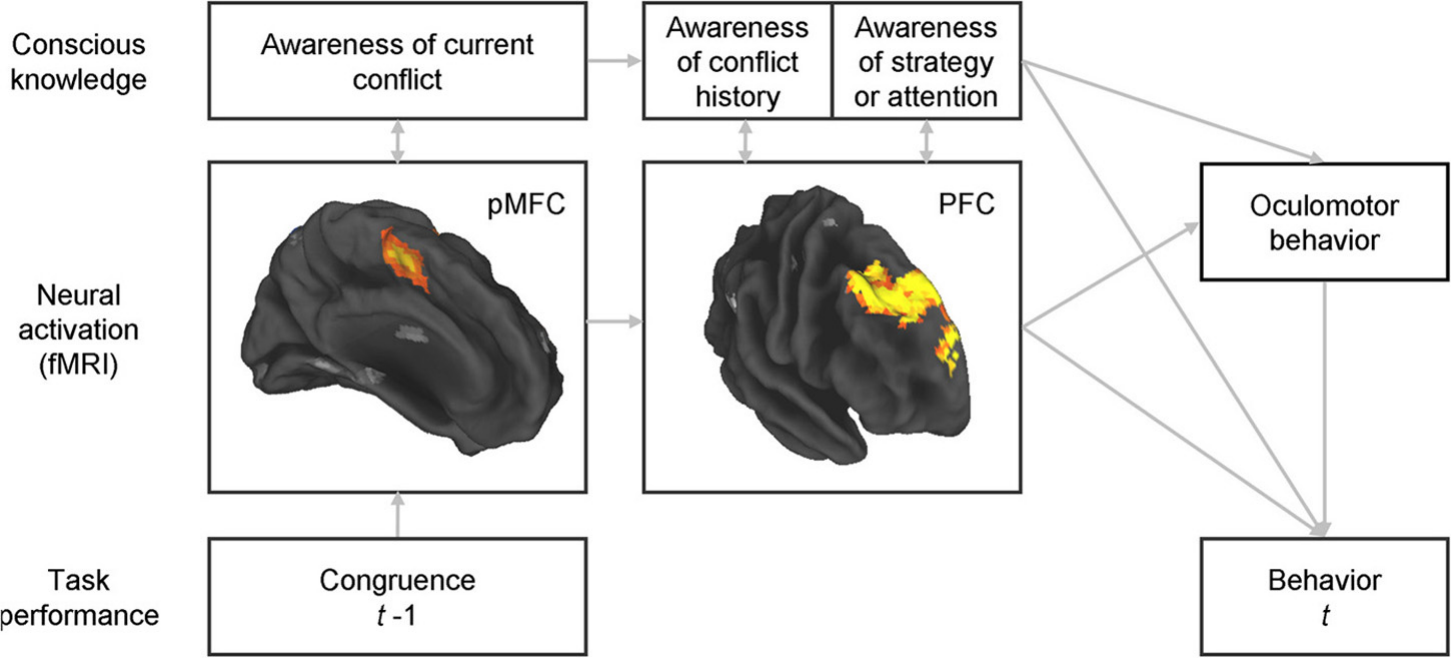
**Integrated assessment of the neural bases of conflict adaptation, potential strategic changes in the allocation of spatial attention, and adaptive changes in behavior, together with assessment of the accompanying conscious knowledge and of whether such knowledge plays a role in strategic changes in spatial attention or in performance adjustments.** Simultaneous, multimodal assessment of brain activity, oculomotor behavior, choice and reaction-time behavior, and conscious knowledge would permit an understanding of the inter-relations between all of these variables. Some questions of particular interest would include: (1) whether awareness of each of the components of control is associated with greater activity in the corresponding brain regions (as predicted by our view on the nature of consciousness); (2) whether adjustments in oculomotor behavior that potentially reflect an expectancy of a certain type of stimulus are associated with conscious knowledge of such expectancy and of its effect on the allocation of spatial attention; and (3) whether behavioral adjustments (of oculomotor behavior or of choice and reaction times) are fully mediated by conscious knowledge or whether instead they can be adaptively influenced by neural activity in the PFC without accompanying conscious knowledge (as predicted by our hypothesis that neural activity in these circuits can be causally efficacious even it is not accompanied by conscious knowledge). pMFC, posterior medial frontal cortex (encompassing the dorsal anterior cingulate cortex and the pre-supplementary motor area); PFC, prefrontal cortex (specifically, rostral dorsomedial, and dorsolateral prefrontal cortex).

The potential role of consciousness in conflict adaptation could be examined by inquiring about participants' knowledge of their past conflict history and their use of strategy at specific time periods during a standard conflict task (Figure 1), and determining the extent to which such knowledge mediates behavioral adaptations. The inter-stimulus interval, for the reasons presented above, may be an appropriate time period for these inquiries. To avoid common failures in reporting conscious knowledge when open-ended questions are used, close-ended questions would be preferred (Maia and McClelland, 2004). The questionnaire should focus on the subcomponents of control that underlie conflict adaptation. At least two aspects of the control mechanism underlying conflict adaptation seem certain: it depends on prior conflict and its engagement benefits performance, i.e., once the control mechanism is engaged it contributes to improve subsequent conflict resolution. Consequently, the questionnaire should target participants' knowledge about the type of stimuli presented on preceding trials and their conscious expectations concerning the upcoming stimulus. Participants may possibly expect repetitions or alternations of certain stimulus types given the preceding sequence, even if stimuli are arranged in a random series (Huettel et al., 2002). The second goal of these inquiries would deal with specific cognitive or behavioral strategies that the individual might deploy in anticipation of the upcoming trial (e.g., focusing spatial attention on a specific region of the screen, preparing an “if-then” strategy, etc.). Lastly, subjective but non-specific sensations such as arousal or attention should also be assessed, as participants might not explicitly know, for example, the history of conflict in the recent trials but nonetheless have a subjective sense that they need to pay, or are paying, more attention following conflict trials.

### Brain-knowledge-behavior analyses

A multimodal approach that includes recordings of neural activity, assessment of conscious knowledge, and behavioral measurements should be used to permit the assessment of the relations between these three variables. Our perspective that conscious and unconscious knowledge may differ mostly in the intensity and duration of neuronal firing predicts that greater neuronal activation measured, for example, with fMRI, should correlate with knowledge that is more conscious. In addition, greater activation should also, naturally, have a greater effect on behavioral adjustments. Thus, to some extent, we expect neuronal activation, conscious knowledge, and behavioral adjustments to be substantially correlated. However, we suggest that even activation that remains below the threshold required to enter consciousness can still be causally efficacious; thus, we predict that behavioral adjustments can occur even in the absence of conscious knowledge. Using path analysis, we recently showed that greater activity in the aforementioned DMPFC-DLPFC system during inter-trial intervals predicted greater behavioral adjustment to conflict on a trial-by-trial basis (Horga et al., 2011). With the measure of conscious knowledge, we could also test whether the effects of activation strength on behavior are mediated by conscious knowledge (Figure 1). The addition of eye tracking to this design, if specific oculomotor behaviors were linked to conflict adaptation, could further unravel the relationships between neural activation, strategic expectancies (reflected in oculomotor behavior), adaptive improvements in performance, and potential conscious knowledge about the strategic expectancies and their influence on behavior. In summary, this multimodal approach would allow us to assess whether, for purposes of conflict adaptation, conscious experiences are epiphenomenal or whether instead they play a central role in mediating the relationship between activity in the regions that have previously been implicated in conflict adaptation and adaptive control of behavior.

## Concluding remarks

Subliminal-priming paradigms have thus far been the method of choice for studying the role of unconscious processing in cognitive control. Despite some early contradictory findings, overall these studies suggest that unconsciously triggered conflict can induce adjustments in control mechanisms. These findings add to others that similarly demonstrate that unconscious processes possess several advanced characteristics (e.g., flexibility) that have traditionally been associated with conscious processes (Wokke et al., 2011). The dichotomy between higher-order control mechanisms that are conscious versus less complex, reflective mechanisms that are unconscious—each of which with its own separate neural substrates and processes—therefore, now seems less appealing than it once did. As an alternative to this idea, we have suggested a more graded view, in which conscious and unconscious processes might rely on the same neural substrates and perform the same processing, differing mostly on the quality of the representation. We have shown that this perspective seamlessly explains the bulk of the literature on unconscious influences in cognitive control.

One limitation of the subliminal-priming approach is that it seeks only to determine whether processes initiated by a subliminally presented prime affect cognitive control. Such an approach is, therefore, uninformative about the potential involvement of conscious versus unconscious processing under more standard task conditions (in which the stimuli themselves are presented supraliminally, but their effect on behavioral adjustments could be mediated by conscious or unconscious processing). We, therefore, proposed a complementary approach that uses standard task conditions but adds a questionnaire to assess participants' conscious knowledge. We indicated how a multi-modal approach could be used to understand the relation between activation in cognitive control areas, conscious knowledge, and behavioral adjustments—including assessing whether conscious knowledge mediates the effect of activation in cognitive control areas on behavioral adjustments. Overall, we hope that both the theoretical perspective that we articulated in this article and our suggestions about a complementary empirical approach to these issues could be of value in guiding future thinking and experimentation in this area.

### Conflict of interest statement

The authors declare that the research was conducted in the absence of any commercial or financial relationships that could be construed as a potential conflict of interest.
